# Evaluation of the Ribosomal Protein S1 Gene (*rpsA*) as a Novel Biomarker for *Mycobacterium* Species Identification

**DOI:** 10.1155/2015/271728

**Published:** 2015-04-05

**Authors:** Hongfei Duan, Guan Liu, Xiaobo Wang, Yuhong Fu, Qian Liang, Yuanyuan Shang, Naihui Chu, Hairong Huang

**Affiliations:** National Clinical Laboratory on Tuberculosis, Beijing Key Laboratory on Drug-Resistant Tuberculosis, Beijing Chest Hospital, Capital Medical University, Beijing Tuberculosis & Thoracic Tumor Research Institute, 97 Beimachang, Tongzhou Qu, Beijing 101149, China

## Abstract

*Objectives*. To evaluate the resolution and reliability of the *rpsA* gene, encoding ribosomal protein S1, as a novel biomarker for mycobacteria species identification. *Methods*. A segment of the *rpsA* gene (565 bp) was amplified by PCR from 42 mycobacterial reference strains, 172 nontuberculosis mycobacteria clinical isolates, and 16 *M. tuberculosis* complex clinical isolates. The PCR products were sequenced and aligned by using the multiple alignment algorithm in the MegAlign package (DNASTAR) and the MEGA program. A phylogenetic tree was constructed by the neighbor-joining method. *Results*. Comparative sequence analysis of the *rpsA* gene provided the basis for species differentiation within the genus *Mycobacterium*. Slow- and rapid-growing groups of mycobacteria were clearly separated, and each mycobacterial species was differentiated as a distinct entity in the phylogenetic tree. The sequences discrepancy was obvious between *M. kansasii* and *M. gastri, M. chelonae* and *M. abscessus, M. avium* and *M. intracellulare*, and *M. szulgai* and *M. malmoense*, which cannot be achieved by 16S ribosomal DNA (rDNA) homologue genes comparison. 183 of the 188 (97.3%) clinical isolates, consisting of 8 mycobacterial species, were identified correctly by *rpsA* gene blast. *Conclusions*. Our study indicates that *rpsA* sequencing can be used effectively for mycobacteria species identification as a supplement to 16S rDNA sequence analysis.

## 1. Introduction

Members of the genus* Mycobacterium* are widespread in nature and range from harmless saprophytic species to strict pathogens that cause serious human and animal diseases. Both slow-growing mycobacteria and rapid-growing mycobacteria can cause human infections. Traditionally, taxonomy based on biochemical characteristics has been used for species determination of mycobacteria, but this approach is limited due to the overlapping biochemical and phenotypic patterns among the different mycobacterial species. Another approach using analysis of cell-wall fatty acid and mycolic acid composition is also limited by profile similarity among some emerging nontuberculosis mycobacteria (NTM) [1]. 16S rDNA homologue gene sequence comparison has been used as an important method for the mycobacterial species identification; however, ambiguous results have been obtained either due to the presence of more than one copy of the 16S rDNA gene within the genome, for example, in* M. celatum* and* M. terrae *complex [2, 3], or due to sequence homology between species [4]. Therefore, alternative phylogenetic markers which are capable of complementing 16S rRNA gene would be useful for the phylogenetic study and species identification of the genus* Mycobacterium*.

Ribosomal protein S1 (*rpsA*), which is in the 30S ribosome subunit, contains the S1 domain that has been found in a large number of RNA-associated proteins.* RpsA* is a vital protein involved in protein translation and the ribosome-sparing process of translation. In addition, it has been reported that* RpsA* is the target of pyrazinoic acid, the active form of the antituberculosis drug pyrazinamide [5].* Mycobacterium tuberculosis* has a single copy of the* rpsA* gene in the genome [6], while the sequence homology among different mycobacteria species in which the gene has already been sequenced is between 86.7 and 100%. This suggests that* rpsA* may be suitable for phylogenetic study of the genus* Mycobacterium*. In this paper, we report an evaluation of the* rpsA* gene as a novel biomarker for mycobacteria species identification.

## 2. Materials and Methods

### 2.1. Mycobacterial Reference Strains and Clinical Isolates

42 type and reference strains of the genus* Mycobacterium* (Table 1), 172 clinical NTM isolates, and 16* M. tuberculosis* complex (MTC) isolates were investigated. All the type and reference strains were purchased from the American Type Culture Collection (ATCC) and all the clinical isolates used in this study were obtained from the Clinical Database and Sample Bank of tuberculosis of Beijing, National Clinical Lab on Tuberculosis, Beijing Chest Hospital. All the clinical NTM isolates were identified to the species level by sequence alignment of at least two of the following: 16S rDNA, 16-23S rRNA gene internal transcribed spacer (ITS), and rpoB and hsp65 genes as described before [2, 7–9]. Following sequencing, the 172 NTM clinical strains were found to include 10 strains of* M. avium*, 75 strains of* M. intracellulare*, 23 strains of* M. kansasii*, 39 strains of* M. abscessus*, 17 strains of* M. fortuitum* complex, 7 strains of* M. gordonae*, and 1 strain of* M. neoaurum*.

### 2.2. *rpsA* Gene Amplification and Sequencing

DNA was released from cultured mycobacteria by boiling the cultured mycobacterial suspension in TE buffer for 10 min. After centrifugation, the supernatant was used for PCR amplification [10]. The primers used were forward primer, 5′-CCCTACATCGGCAAGGAG-3′, position 487–504 in the* rpsA* gene of* Mycobacterium tuberculosis*, GenBank accession number NC_000962.2, and reverse primer, 5′-TGTCGATGACCTTGACCATC-3′, position 1032–1051 in the* rpsA* gene of* Mycobacterium tuberculosis*, GenBank accession number NC_000962.2. The amplified product was 565 bp. PCR products were purified and sequenced by a commercial company (YINGJUN Biotech Company, Beijing, China) using ABI 3730 DNA Analyzer (Applied Biosystems, California, USA).

### 2.3. Sequence Analysis and the Phylogenetic Tree Constructions

In addition to the 42 reference strains, the* rpsA* sequences of 5 other mycobacterial species were obtained directly from GenBank, including* M. avium* subspecies* paratuberculosis* (GenBank accession number NC_002944.2),* M. ulcerans* (GenBank accession number NC_008611.1),* M. vanbaalenii* (GenBank accession number NC_008726.1),* M. abscessus* subspecies* massiliense* (GenBank accession number NC_018150.1), and* M. canettii* (GenBank accession number NC_015848.1). All sequences were aligned and homology was calculated by using the multiple alignment algorithm in the MegAlign package (Windows version 7.1.0; DNASTAR, Madison, Wis). 527 bp of sequence (excluding 38 nucleotides at each end of the amplicon, corresponding to the primer binding sites) was analyzed for the phylogenetic tree construction by the neighbor-joining method using the MEGA 5.05 package (http://www.megasoftware.net/mega.php). A bootstrap analysis (1000 repeats) using* Rhodococcus equi* (GenBank accession number NC_006361.1) as the outgroup was performed to evaluate the topology of the phylogenetic tree.

### 2.4. Species Identification of the Clinical Isolates

The 188 clinical isolates were analyzed blindly. Sequences, minus the known PCR primer sequences, were assembled and edited by using SeqMan software (version 7.1.0; DNASTAR, Madison, WI). Isolates were identified by comparing sequences by using a FASTA BLASTn search with MegAlign (version 7.1.0; DNASTAR, Madison, WI) to an in-house database of sequences consisting of type and reference strains from external culture collections.

## 3. Results

### 3.1. *rpsA* Sequence Alignment of the Reference Strains

Between 85.4% and 100% sequence homology (interspecies divergence, 0% to 14.6%) was observed among the 42 tested reference strains and the 5 additional mycobacterial species whose sequences were obtained from the GenBank database (Table 2). All the* rpsA* gene sequences of the analyzed mycobacteria strains were distinct from the outgroup strain* R. equi*. Among the 19 reference strains of the slow-growing* Mycobacterium* genus, 11 strains were greater than 97% homology, including 5* M. tuberculosis* complex strains. Among the 28 reference strains of the rapid-growing* Mycobacterium* genus, 14 strains were greater than 97% homology (Table 1). The pathogenic* M. kansasii* was easily differentiated from the nonpathogenic* M. gastri* (95.8% homology), while those two species were not distinguishable by the 16S rDNA sequence alignment. The sequence homologies between various species were 91.8% between* M. chelonae* and* M. abscessus*, 95.6% between* M. avium* and* M. intracellulare,* and 93.9% between* M. szulgai* and* M. malmoense*. However, the sequence homology between other species was higher; for example, it was 99.6% between* M. ulcerans* and* M. marinum* and 98.7% between* M. abscessus* subspecies* massiliense* and* M. abscessus*. All members of the* M. tuberculosis* complex had identical sequences as did* M. senegalense* and* M. thermoresistibile*,* M. parafortuitum* and* M. trivial*,* M. diernhoferi* and* M. duvalii*, and* M. austroafricanum* and* M. terrae*.

### 3.2. Phylogenetic Tree Construction

**Figure 1:**
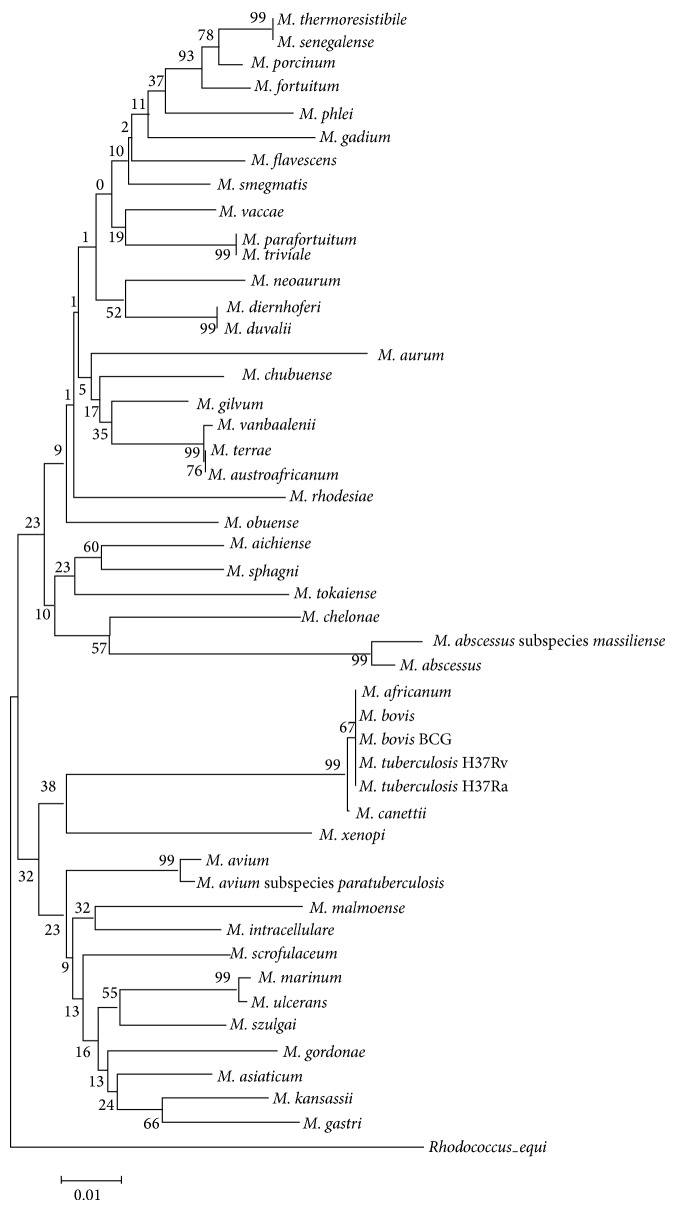
Phylogenetic tree based on* rpsA* gene sequences shows the relationship of the 47 type strains of mycobacteria and 1 outgroup strain. This tree was constructed by the neighbor-joining method. Topology was also evaluated by bootstrap analysis (MEGA program, 1000 repeats, with* R. equi* as the outgroup). The numerical values in the tree represent bootstrap results. The distance between two strains is the sum of the branch lengths between them.

A phylogenetic tree, which provided the basis for species differentiation in the genus* Mycobacterium*, was constructed (Figure 1). The absolute majority tested species showed good separation. The rapid-growing species were well defined from the slow-growing species in the tree.* M. chelonae*,* M. abscessus* subspecies* massiliense,* and* M. abscessus*, which are categorized in the pathogenic taxonomic group of rapid-growing mycobacteria, formed a distinctive cluster which was much closer to the slow-growing species compared with the nonpathogenic group of rapid-growing mycobacteria. The reliability of the phylogenetic tree was verified by the bootstrap method, using* R. equi* as the outgroup.

### 3.3. Species Identification Outcomes of the Clinical Isolates

*rpsA* sequence and alignment efficiently identified clinical isolates representing 8 mycobacterial species. We found that a criterion of first distinct* rpsA* sequence match >97% confirmed the identification of 183 of 188 (97.3%) clinical isolates. 20* M. abscessus* strains, 2* M. fortuitum* strains, and 10* M. avium* strains were identified to the subspecies level. Identification discrepancies between* rpsA* and other sequences were only encountered with 4* M. gordonae* strains and 1* M. kansasii* strain (see Table 3). No discrepancies were found in the species submitted as* M. intracellulare*,* M. avium*,* M. abscessus*, and* M. tuberculosis* complex.

The sequence divergence among MTC members was 0.4%. The intraspecies divergence among the 188 clinical strains ranged from 0.6% to 5.3% (Table 3). The sequence diversity among the* M. abscessus* was 0.6%, while that among the* M. gordonae* clinical isolates was 5.3%. No* M. gordonae* clinical strain had an identical sequence with that of the* M. gordonae* reference strain (ATCC14470). Interestingly, the* rpsA* sequence of one strain among the 23* M. kansasii* isolates exhibited a relatively low level of sequence similarity (95.6%) to that of the reference strain (ATCC 12478), while those of all the other* M. kansasii* isolates were identical. Since the strain was confirmed as* M. kansasii* by* rpoB* and* hsp65* gene alignment, it might suggest a distant variant or a new subtype.

## 4. Discussion

16S rDNA gene sequence alignment has been used as the reference method for mycobacterial species identification. However, it has been reported that, by using the 16S rDNA gene alone for species identification of clinical NTM, 37% of such isolates remained unclassified which illustrates the need for additional molecular tools for proper phylogenetic assignment and accurate NTM identification [11]. The common assumption that bacterial isolates belong to the same species if they have fewer than 5–15 bp differences within the 16S rDNA gene sequence [12] or if they have more than 97% 16S rDNA gene sequence identities [13] may not be applicable to genus of* Mycobacterium*, whose members are much more closely related to each other. Furthermore, some mycobacterial strains shared uniform sequence of 16S rDNA gene, such as* M. intracellulare* and* M. avium*,* M. kansasii* and* M. gastri*,* M. abscessus* and* M. chelonae*, and* M. szulgai* and* M. malmoense*.

In this study, we found that the* rpsA* gene is useful as a complementary method to the 16S rDNA for mycobacterial species identification. Among the 47 reference strains and totally 1081 paired comparisons {1081 = [*na*_1_ + *n*(*n* − 1)*d*/2]; *a*_1_ = 1, *n* = 46, *d* = 1}, the* rpsA* gene alone can differentiate 97.2% (1051 out of 1081) of them if using 97% sequence homology as cutoff value. When referring to the phylogenetic tree, the* rpsA* resolution might increase. For the most commonly seen pathogenic NTM species such as* M. intracellulare, M. avium, M. abscessus, M. chelonae,* and* M. xenopi*,* rpsA* gene sequence could differentiate these species easily. Furthermore, 183 of the 188 (97.3%) clinical isolates, representing 8 mycobacterial species, were identified correctly by* rpsA* gene blast. Discrepancies of identification were only encountered with 4* M. gordonae* strains and 1* M. kansasii* strain. Both these species have reportedly more intraspecies sequence divergence [9, 14–16]. Three out of the 4* M. gordonae* strains with discrepant identification still had the first distinction as* M. gordonae* by* rpsA,* but the sequence identity was lower than 97%, which means the identification might be correct when a more complete in-house database is being developed.

*rpsA* alone cannot differentiate the following: between members of the* M. tuberculosis* complex, between* M. senegalense* and* M. thermoresistibile*, between* M. parafortuitum* and* M. trivial*, between* M. diernhoferi* and* M. duvalii*, and between* M. austroafricanum* and* M. terrae*; however, these species are also difficult to be separated by other markers alone such as 16s rDNA,* ITS*,* rpoB,* and hsp65. Several reference strains had similar homology with two or more species which suggests inadequate taxonomy within the currently described species. Based on nucleotide sequences of* rpoB, hsp65,* and* sodA* in clinical isolates of the* Mycobacterium abscessus* group, one-fourth of isolates had discordant identification [16]. Multilocus sequence typing and sequence analysis of several genes facilitate the identification of closely related species or subspecies [17].

In our study, we have demonstrated that the* rpsA* homology gene is a promising marker for mycobacterial species identification for both fast- and slow-growing mycobacteria. To be a good marker for species differentiation, the target gene should be stable and sequence variations should occur randomly; additionally, an extremely conserved or highly variable gene may not be adequate. As a single-copy house-keeping gene,* rpsA* gene could work well as a target gene for* Mycobacterium* species discrimination without ambiguous identification. The 16S rDNA gene has higher homology within the mycobacteria compared to the rpsA gene: 94.3% to 100% compared to 85.4% to 100%. According to our work, when used as sole marker,* rpsA* had better resolution than* 16s rDNA* but had similar resolution as* ITS, rpoB,* and* hsp65*.

Due to the high resolution power of* rpsA* for species identification, we presume it has potential of clinical use. From our own experience, we recommend that when performing species identification by homologue DNA sequence comparison, one should start with 16s RNA gene, plus at least one of the other markers such as* ITS, rpoB, hsp65,* and* rpsA*. When conflicting outcome or dubious outcomes yield, more markers should be added further. Even though 16s RNA gene is inferior to the other markers considering the capacity, it should be chosen firstly since 16s RNA gene has the most robust sequence database, which can help to avoid big identification errors due to unreliable database of other markers [18].

The main deterrents for the primary use of* rpsA* sequencing as a routine means of identifying mycobacteria reside in the need for a comprehensive database. Therefore, we constructed our own* rpsA* sequence database by including as many type strains as possible and integrating* rpsA* sequences deposited in GenBank such as* M. avium* subspecies* paratuberculosis*,* M. ulcerans, M. vanbaalenii, M. abscessus* subspecies* massiliense,* and* M. canettii*. Besides type strains,* rpsA* sequences of confirmed clinical strains were also included. We will constantly upgrade our database and the capability to identify the most recent described species.

## Figures and Tables

**Table 1:** Percentage *rpsA* sequence match among type strain.

Species	Type strain	First match (%)^a^	Second match (%)^a^
*M. thermoresistibile *	ATCC19527	*M. senegalense* (100%)	*M. porcinum* (98.7%)
*M. senegalense *	ATCC35796	*M. thermoresistibile* (100%)	*M. porcinum* (98.7%)
*M. porcinum *	ATCC33776	*M. senegalense/M. thermoresistibile* (98.7%)	*M. fortuitum* (98.3%)
*M. fortuitum *	ATCC6481	*M. porcinum* (98.3%)	*M. senegalense/M. thermoresistibile* (98.1%)
*M. phlei *	ATCC11758	*M. porcinum* (96.5%)	*M. fortuitum* (96.1%)
*M. gadium *	ATCC27726	*M. porcinum/M. fortuitum* (95.7%)	*M. smegmatis* (95.3%)
*M. flavescens *	ATCC14474	*M. smegmatis* (96.6%)	*M. vaccae* (96.0%)
*M. smegmatis *	ATCC19420	*M. vaccae/M. parafortuitum/M. triviale* (96.6%)	*M. senegalense/M. thermoresistibile* (96.3%)
*M. vaccae *	ATCC15483	*M. diernhoferi/M. duvalii* (96.8%)	*M. parafortuitum/M. triviale* (96.6%)
*M. parafortuitum *	ATCC19686	*M. triviale* (100%)	*M. terrae/M. austroafricanum* (96.6%)
*M. triviale *	ATCC23292	*M. parafortuitum* (100%)	*M. terrae/M. austroafricanum* (96.6%)
*M. neoaurum *	ATCC25795	*M. diernhoferi/M. duvalii* (96.4%)	*M. porcinum* (95.5%)
*M. diernhoferi *	ATCC19340	*M. duvalii* (100%)	*M. vaccae* (96.8%)
*M. duvalii *	ATCC43910	*M. diernhoferi* (100%)	*M. vaccae* (96.8%)
*M. aurum *	ATCC23366	*M. gilvum* (93.9%)	*M. chubuense/M. vanbaalenii* (93.0%)
*M. chubuense *	ATCC27278	*M. gilvum* (96.6%)	*M. vanbaalenii* (96.0%)
*M. gilvum *	ATCC43909	*M. vanbaalenii* (97.2%)	*M. terrae/M. austroafricanum* (97.0%)
*M. vanbaalenii *	PYR-1	*M. terrae/M. austroafricanum* (99.8%)	*M. gilvum* (97.2%)
*M. terrae *	ATCC15755	*M. austroafricanum* (100%)	*M. vanbaalenii* (99.8%)
*M. austroafricanum *	ATCC33464	*M. terrae* (100%)	*M. vanbaalenii* (99.8%)
*M. rhodesiae *	ATCC27024	*M. terrae/M. austroafricanum* (93.9%)	*M. obuense* (93.7%)
*M. obuense *	ATCC27023	*M. porcinum /M. fortuitum* (96.1%)	*M. senegalense/M. thermoresistibile* (96.5%)
*M. aichiense *	ATCC27280	*M. sphagni* (95.8%)	*M. vaccae* (94.7%)
*M. sphagni *	ATCC33027	*M. aichiense* (95.8%)	*M. porcinum* (95.5%)
*M. tokaiense *	ATCC27282	*M. porcinum* (95.3%)	*M. smegmatis* (93.7%)
*M. chelonae *	ATCC35752	*M. obuense* (93.9%)	*M. phlei* (93.5%)
*M. abscessus *subspecies* massiliense *	str. GO 06	*M. abscessus* (98.7%)	*M. sphagni* (91.4%)
*M. abscessus *	ATCC19977	*M. abscessus* subspecies *massiliense* (98.7%)	*M. chubuense* (92.0%)
*M. tuberculosis *H37Rv	ATCC27294	*M. tuberculosis *H37Ra*/M. africanum/M. bovis *BCG*/M. bovis* (100%)	*M. canettii* (99.8%)
*M. tuberculosis *H37Ra	ATCC25177	*M. tuberculosis* H37Rv*/M. africanum/M. bovis *BCG*/M. bovis* (100%)	*M. canettii* (99.8%)
*M. africanum *	ATCC25420	*M. tuberculosis* H37Ra */M. tuberculosis* Rv/*M. bovisBCG/M. bovis* (100%)	*M. canettii* (99.8%)
*M. bovis *BCG	ATCC35735	*M. tuberculosis* H37Ra */M. tuberculosis* H37Rv */M. africanum/M. bovis* (100%)	*M. canettii* (99.8%)
*M. bovis *	ATCC19210	*M. tuberculosis* H37Ra*/M. tuberculosis *H37Rv*/M. africanum/M. bovis *BCG (100%)	*M. canettii* (99.8%)
*M. canettii *	CIPT140010059	*M. tuberculosis/M. africanum/M. bovis *BCG*/M. bovis* (99.8%)	*M. asiaticum* (93.2%)
*M. xenopi *	ATCC19250	*M. asiaticum* (93.0%)	*M. avium *subspecies* paratuberculosis* (92.4%)
*M. avium *	ATCC25291	*M. avium* subspecies *paratuberculosis* (99.6%)	*M. asiaticum* (95.8%)
*M. avium *subspecies* paratuberculosis *	K-10	*M. avium* (99.4%)	*M. asiaticum* (96%)
*M. malmoense *	ATCC29571	*M. intracellulare *(94.3%)	*M. szulgai* (93.9%)
*M. intracellulare *	ATCC13950	*M. avium* (95.6%)	*M. avium *subspecies* paratuberculosis* (95.4%)
*M. scrofulaceum *	ATCC19981	*M. asiaticum* (95.4%)	*M. szulgai* (95.1%)
*M. marinum *	ATCC927	*M. ulcerans* (99.6%)	*M. szulgai* (96.0%)
*M. ulcerans *	Agy99	*M. marinum* (99.6%)	*M. szulgai* (96.0%)
*M. szulgai *	ATCC25799	*M. marinum/M. ulcerans* (96.0%)	*M. asiaticum* (95.6%)
*M. gordonae *	ATCC14470	*M. asiaticum* (95.3%)	*M. kansasii* (95.1%)
*M. asiaticum *	ATCC25276	*M. simiae* (97.7%)	*M. kansasii* (96.4%)
*M. kansasii *	ATCC12478	*M. asiaticum* (96.3%)	*M. gastri* (95.8%)
*M. gastri *	ATCC15754	*M. kansasii* (95.8%)	*M. asiaticum* (94.5%)
*Rhodococcus equi *	103S	*M. smegmatis* (90.3%)	*M. intracellulare* (90.1%)

^a^Beside the queried type strain itself, the first matched or the second matched type strain.

**Table 2:** Sequence pair distances of 47 type strains of mycobacteria and 1 out-group strain determined byusing the multiple alignment algorithm in the MegAlign package (version 7.1.0; DNASTAR, Madison, WI).

Percent identity																																																		
	1	2	3	4	5	6	7	8	9	10	11	12	13	14	15	16	17	18	19	20	21	22	23	24	25	26	27	28	29	30	31	32	33	34	35	36	37	38	39	40	41	42	43	44	45	46	47	48		
1	—	100.0	98.7	98.1	96.0	95.3	95.4	96.4	96.2	94.5	94.5	95.1	94.5	94.5	92.0	93.9	94.1	94.3	94.5	94.5	92.8	95.6	93.7	94.7	93.2	92.4	88.8	89.2	91.1	91.1	91.1	91.1	91.1	91.3	91.5	93.9	94.1	91.3	93.0	92.8	92.6	92.6	92.4	93.4	93.4	92.4	92.8	89.2	1	*M. thermoresistibile *
2	0.0	—	98.7	98.1	96.0	95.3	95.4	96.4	96.2	94.5	94.5	95.1	94.5	94.5	92.0	93.9	94.1	94.3	94.5	94.5	92.8	95.6	93.7	94.7	93.2	92.4	88.8	89.2	91.1	91.1	91.1	91.1	91.1	91.3	91.5	93.9	94.1	91.3	93.0	92.8	92.6	92.6	92.4	93.4	93.4	92.4	92.8	89.2	2	*M. senegalense *
3	1.3	1.3	—	98.3	96.6	95.8	95.8	96.8	96.8	94.7	94.7	95.6	95.4	95.4	92.0	94.1	95.4	94.9	95.1	95.1	93.0	96.2	94.7	95.6	94.5	93.0	89.4	89.8	91.1	91.1	91.1	91.1	91.1	91.3	92.0	94.5	94.7	92.0	93.4	93.5	93.2	93.2	92.8	93.7	93.7	93.0	92.8	89.4	3	*M. porcinum *
4	1.9	1.9	1.7	—	96.2	95.8	95.8	96.2	96.2	94.1	94.1	95.4	94.5	94.5	92.0	94.3	94.5	94.3	94.5	94.5	93.9	96.2	94.9	94.9	93.7	93.7	90.1	90.5	90.9	90.9	90.9	90.9	90.9	91.1	92.0	94.7	94.9	91.3	93.2	93.2	93.0	93.4	92.8	93.5	93.7	92.8	93.0	89.2	4	*M. fortuitum *
5	4.1	4.1	3.5	3.9	—	94.3	95.4	96.4	96.0	93.9	93.9	93.4	94.3	94.3	91.5	94.3	94.3	94.5	94.7	94.7	91.5	94.3	94.1	94.9	92.8	91.1	89.8	90.3	90.1	90.1	90.1	90.1	90.1	90.3	91.1	93.5	93.7	91.3	93.4	93.0	93.5	93.5	93.0	93.7	93.4	93.0	92.0	89.6	5	*M. phlei *
6	4.9	4.9	4.3	4.3	5.9	—	95.1	95.3	94.1	93.7	93.7	94.7	94.1	94.1	91.3	93.7	93.5	93.4	93.5	93.5	93.2	93.7	93.7	93.7	93.0	92.4	88.6	89.0	90.7	90.7	90.7	90.7	90.7	90.9	91.5	94.3	94.1	90.7	93.2	92.0	91.8	92.2	91.5	93.0	93.2	91.8	91.5	88.0	6	*M. gadium *
7	4.7	4.7	4.3	4.3	4.7	5.1	—	96.6	96.0	95.4	95.4	94.7	94.7	94.7	93.0	95.1	95.3	95.6	95.8	95.8	93.2	94.1	94.9	94.7	92.4	92.0	90.1	90.5	92.4	92.4	92.4	92.4	92.4	92.6	92.8	94.9	94.7	93.0	94.1	94.9	93.7	93.7	93.5	93.9	94.3	92.6	93.4	90.3	7	*M. flavescens *
8	3.7	3.7	3.3	3.9	3.7	4.9	3.5	—	96.6	96.6	96.6	95.3	96.2	96.2	92.0	96.2	96.2	96.4	96.2	96.2	93.5	94.7	94.5	94.5	93.9	91.5	90.5	90.9	91.1	91.1	91.1	91.1	91.1	91.3	92.2	93.7	93.5	92.0	94.3	93.5	93.2	93.2	93.0	93.4	93.5	92.4	92.2	90.9	8	*M. smegmatis *
9	3.9	3.9	3.3	3.9	4.1	6.1	4.1	3.5	—	96.6	96.6	95.1	96.8	96.8	92.4	95.8	95.6	96.2	96.4	96.4	93.0	95.6	94.9	94.7	92.4	92.0	91.5	91.8	90.9	90.9	90.9	90.9	90.9	91.1	92.2	94.3	94.5	92.4	93.9	92.8	93.4	93.4	93.2	93.9	93.5	92.8	92.8	89.2	9	*M. vaccae *
10	5.7	5.7	5.5	6.1	6.3	6.5	4.7	3.5	3.5	—	100.0	94.9	96.2	96.2	93.0	94.9	95.8	96.4	96.6	96.6	93.0	94.3	92.8	93.0	92.4	91.7	89.8	90.1	89.9	89.9	89.9	89.9	89.9	90.1	91.5	92.6	92.4	91.3	93.2	92.0	91.1	91.1	91.3	91.8	91.5	90.3	91.7	89.0	10	*M. parafortuitum *
11	5.7	5.7	5.5	6.1	6.3	6.5	4.7	3.5	3.5	0.0	—	94.9	96.2	96.2	93.0	94.9	95.8	96.4	96.6	96.6	93.0	94.3	92.8	93.0	92.4	91.7	89.8	90.1	89.9	89.9	89.9	89.9	89.9	90.1	91.5	92.6	92.4	91.3	93.2	92.0	91.1	91.1	91.3	91.8	91.5	90.3	91.7	89.0	11	*M. triviale *
12	5.1	5.1	4.5	4.7	7.0	5.5	5.5	4.9	5.1	5.3	5.3	—	96.4	96.4	91.8	94.5	95.3	95.1	95.3	95.3	94.1	95.1	92.8	92.2	92.6	93.0	90.3	90.7	89.9	89.9	89.9	89.9	89.9	90.1	91.3	93.2	92.8	89.9	92.0	92.0	91.7	91.7	91.1	91.8	92.4	90.9	89.9	88.2	12	*M. neoaurum *
13	5.7	5.7	4.7	5.7	5.9	6.1	5.5	3.9	3.3	3.9	3.9	3.7	—	100.0	92.6	95.3	95.8	95.6	95.8	95.8	94.1	94.9	94.1	93.7	92.8	92.2	91.3	91.7	89.4	89.4	89.4	89.4	89.4	89.6	90.7	92.0	91.8	91.1	93.0	91.8	91.5	91.5	91.3	91.7	91.8	91.3	90.5	89.8	13	*M. diernhoferi *
14	5.7	5.7	4.7	5.7	5.9	6.1	5.5	3.9	3.3	3.9	3.9	3.7	0.0	—	92.6	95.3	95.8	95.6	95.8	95.8	94.1	94.9	94.1	93.7	92.8	92.2	91.3	91.7	89.4	89.4	89.4	89.4	89.4	89.6	90.7	92.0	91.8	91.1	93.0	91.8	91.5	91.5	91.3	91.7	91.8	91.3	90.5	89.8	14	*M. duvalii *
15	8.4	8.4	8.4	8.4	9.1	9.3	7.4	8.5	8.0	7.4	7.4	8.6	7.8	7.8	—	93.0	93.9	93.0	93.2	93.2	91.7	91.8	91.1	91.1	89.4	91.1	89.8	90.1	89.2	89.2	89.2	89.2	89.2	89.4	88.8	90.1	90.5	91.5	90.5	90.5	89.6	89.8	89.8	89.2	90.1	89.6	89.0	87.7	15	*M. aurum *
16	6.3	6.3	6.1	5.9	5.9	6.5	5.1	3.9	4.3	5.3	5.3	5.7	4.9	4.9	7.4	—	96.6	96.0	95.8	95.8	93.9	94.7	94.7	93.5	92.8	92.4	91.8	92.4	90.5	90.5	90.5	90.5	90.5	90.7	91.7	93.2	93.0	91.1	92.6	92.4	92.6	93.0	91.3	92.4	93.5	91.5	90.9	88.4	16	*M. chubuense *
17	6.1	6.1	4.7	5.7	5.9	6.8	4.9	3.9	4.5	4.3	4.3	4.9	4.3	4.3	6.3	3.5	—	97.2	97.0	97.0	94.1	95.6	94.5	94.1	93.5	93.4	91.3	91.7	90.3	90.3	90.3	90.3	90.3	90.5	91.3	93.0	93.2	92.0	92.8	93.2	92.6	93.0	91.7	92.8	92.8	91.5	91.1	90.1	17	*M. gilvum *
18	5.9	5.9	5.3	5.9	5.7	7.0	4.5	3.7	3.9	3.7	3.7	5.1	4.5	4.5	7.4	4.1	2.9	—	99.8	99.8	94.1	94.9	92.8	93.4	92.6	93.0	90.7	91.1	90.1	90.1	90.1	90.1	90.1	90.3	91.3	92.8	92.6	91.3	92.4	92.8	92.0	92.0	91.3	92.0	92.4	91.3	92.0	89.2	18	*M. vanbaalenii *
19	5.7	5.7	5.1	5.7	5.5	6.8	4.3	3.9	3.7	3.5	3.5	4.9	4.3	4.3	7.2	4.3	3.1	0.2	—	100.0	93.9	95.1	93.0	93.5	92.8	93.2	90.9	91.3	90.3	90.3	90.3	90.3	90.3	90.5	91.5	93.0	92.8	91.5	92.6	93.0	92.2	92.2	91.5	92.2	92.6	91.5	92.2	89.0	19	*M. terrae *
20	5.7	5.7	5.1	5.7	5.5	6.8	4.3	3.9	3.7	3.5	3.5	4.9	4.3	4.3	7.2	4.3	3.1	0.2	0.0	—	93.9	95.1	93.0	93.5	92.8	93.2	90.9	91.3	90.3	90.3	90.3	90.3	90.3	90.5	91.5	93.0	92.8	91.5	92.6	93.0	92.2	92.2	91.5	92.2	92.6	91.5	92.2	89.0	20	*M. austroafricanum *
21	7.6	7.6	7.4	6.3	9.1	7.2	7.2	6.7	7.4	7.4	7.4	6.1	6.1	6.1	8.9	6.3	6.1	6.1	6.3	6.3	—	93.7	91.8	91.7	91.1	92.6	88.8	89.2	89.4	89.4	89.4	89.4	89.4	89.6	90.3	91.5	91.7	90.1	90.9	90.9	89.9	90.1	89.6	90.7	91.3	90.9	89.9	89.6	21	*M. rhodesiae *
22	4.5	4.5	3.9	3.9	5.9	6.6	6.1	5.5	4.5	5.9	5.9	5.1	5.3	5.3	8.7	5.5	4.5	5.3	5.1	5.1	6.5	—	93.7	94.5	93.4	94.1	90.3	90.7	90.3	90.3	90.3	90.3	90.3	90.5	90.5	93.7	93.9	90.3	92.6	92.8	92.2	92.2	91.8	92.6	93.0	91.7	90.9	89.2	22	*M. obuense *
23	6.5	6.5	5.5	5.3	6.1	6.5	5.3	5.7	5.3	7.6	7.6	7.6	6.1	6.1	9.5	5.5	5.7	7.6	7.4	7.4	8.6	6.5	—	95.8	93.9	93.0	91.7	92.0	90.9	90.9	90.9	90.9	90.9	91.1	92.2	93.7	93.9	91.7	93.2	92.6	93.4	93.7	92.2	93.7	93.9	92.4	91.7	89.0	23	*M. aichiense *
24	5.5	5.5	4.5	5.3	5.3	6.5	5.5	5.7	5.5	7.4	7.4	8.2	6.5	6.5	9.5	6.7	6.1	7.0	6.7	6.7	8.9	5.7	4.3	—	93.7	93.4	91.3	91.8	91.5	91.5	91.5	91.5	91.5	91.7	91.5	93.7	94.1	92.2	93.0	93.5	92.8	93.0	93.0	93.4	94.1	93.2	92.4	89.2	24	*M. sphagni *
25	7.2	7.2	5.7	6.5	7.6	7.4	8.0	6.3	8.0	8.0	8.0	7.8	7.6	7.6	11.5	7.6	6.7	7.8	7.6	7.6	9.5	7.0	6.3	6.5	—	92.0	88.8	89.4	89.6	89.6	89.6	89.6	89.6	89.8	90.5	94.5	94.5	90.7	93.0	92.8	91.8	92.2	92.0	92.4	93.4	92.2	91.5	88.2	25	*M. tokaiense *
26	8.0	8.0	7.4	6.5	9.5	8.0	8.4	9.1	8.4	8.9	8.9	7.4	8.2	8.2	9.5	8.0	7.0	7.4	7.2	7.2	7.8	6.1	7.4	7.0	8.4	—	91.5	91.8	89.6	89.6	89.6	89.6	89.6	89.8	89.8	92.2	92.6	89.4	90.5	91.1	90.7	91.1	89.8	92.0	91.7	90.7	90.3	87.1	26	*M. chelonae *
27	12.1	12.1	11.5	10.6	11.0	12.4	10.6	10.1	9.1	11.0	11.0	10.4	9.3	9.3	11.0	8.6	9.3	9.9	9.7	9.7	12.1	10.4	8.9	9.3	12.1	9.1	—	98.7	86.7	86.7	86.7	86.7	86.7	86.9	88.4	88.4	88.8	88.4	88.6	89.4	89.6	89.6	88.4	90.1	89.8	88.2	87.1	87.1	27	*M. abscessus *
28	11.7	11.7	11.0	10.1	10.4	11.9	10.1	9.7	8.6	10.6	10.6	9.9	8.9	8.9	10.6	8.0	8.9	9.5	9.3	9.3	11.7	9.9	8.4	8.6	11.5	8.7	1.3	—	87.5	87.5	87.5	87.5	87.5	87.7	88.8	88.8	89.2	88.8	89.0	89.8	89.9	89.9	89.0	90.5	90.1	88.6	87.5	87.7	28	*M. abscessus *
29	9.5	9.5	9.5	9.7	10.6	10.0	8.0	9.5	9.7	10.8	10.8	10.8	11.5	11.5	11.7	10.1	10.4	10.6	10.4	10.4	11.5	10.4	9.7	9.1	11.3	11.3	14.6	13.7	—	100.0	100.0	100.0	100.0	99.8	90.9	91.7	92.0	90.9	91.7	92.2	92.0	91.8	92.0	92.8	93.4	92.2	91.8	88.0	29	*M. tuberculosis *
30	9.5	9.5	9.5	9.7	10.6	10.0	8.0	9.5	9.7	10.8	10.8	10.8	11.5	11.5	11.7	10.1	10.4	10.6	10.4	10.4	11.5	10.4	9.7	9.1	11.3	11.3	14.6	13.7	0.0	—	100.0	100.0	100.0	99.8	90.9	91.7	92.0	90.9	91.7	92.2	92.0	91.8	92.0	92.8	93.4	92.2	91.8	88.0	30	*M. tuberculosis *
31	9.5	9.5	9.5	9.7	10.6	10.0	8.0	9.5	9.7	10.8	10.8	10.8	11.5	11.5	11.7	10.1	10.4	10.6	10.4	10.4	11.5	10.4	9.7	9.1	11.3	11.3	14.6	13.7	0.0	0.0	—	100.0	100.0	99.8	90.9	91.7	92.0	90.9	91.7	92.2	92.0	91.8	92.0	92.8	93.4	92.2	91.8	88.0	31	*M. bovis *
32	9.5	9.5	9.5	9.7	10.6	10.0	8.0	9.5	9.7	10.8	10.8	10.8	11.5	11.5	11.7	10.1	10.4	10.6	10.4	10.4	11.5	10.4	9.7	9.1	11.3	11.3	14.6	13.7	0.0	0.0	0.0	—	100.0	99.8	90.9	91.7	92.0	90.9	91.7	92.2	92.0	91.8	92.0	92.8	93.4	92.2	91.8	88.0	32	*M. africanum *
33	9.5	9.5	9.5	9.7	10.6	10.0	8.0	9.5	9.7	10.8	10.8	10.8	11.5	11.5	11.7	10.1	10.4	10.6	10.4	10.4	11.5	10.4	9.7	9.1	11.3	11.3	14.6	13.7	0.0	0.0	0.0	0.0	—	99.8	90.9	91.7	92.0	90.9	91.7	92.2	92.0	91.8	92.0	92.8	93.4	92.2	91.8	88.0	33	*M. bovis *
34	9.3	9.3	9.3	9.5	10.4	9.7	7.8	9.3	9.5	10.6	10.6	10.6	11.2	11.2	11.5	9.9	10.2	10.4	10.2	10.2	11.2	10.2	9.5	8.9	11.1	11.0	14.4	13.5	0.2	0.2	0.2	0.2	0.2	—	91.1	91.8	92.2	90.7	91.8	92.4	91.8	91.7	91.8	92.6	93.2	92.0	91.7	88.0	34	*M. canettii *
35	9.1	9.1	8.4	8.4	9.5	9.1	7.6	8.2	8.2	9.1	9.1	9.3	9.9	9.9	12.2	8.9	9.3	9.3	9.1	9.1	10.4	10.2	8.2	9.1	10.2	11.0	12.6	12.1	9.8	9.8	9.8	9.8	9.8	9.5	—	92.2	92.4	90.7	91.7	92.0	91.1	91.1	90.5	91.8	93.0	91.5	90.9	87.7	35	*M. xenopi *
36	6.3	6.3	5.7	5.5	6.7	5.9	5.3	6.5	5.9	7.8	7.8	7.2	8.4	8.4	10.6	7.2	7.4	7.6	7.4	7.4	9.1	6.5	6.5	6.5	5.7	8.2	12.6	12.1	8.9	8.9	8.9	8.9	8.9	8.7	8.2	—	99.4	93.0	95.6	94.7	93.9	94.3	94.1	95.1	95.8	94.7	93.7	88.6	36	*M. avium *
37	6.1	6.1	5.5	5.3	6.5	6.1	5.5	6.7	5.7	8.0	8.0	7.6	8.6	8.6	10.1	7.4	7.2	7.8	7.6	7.6	8.9	6.3	6.3	6.1	5.7	7.8	12.1	11.7	8.4	8.4	8.4	8.4	8.4	8.2	8.0	0.6	—	93.5	95.4	95.1	94.5	94.9	94.5	95.4	96.0	95.3	93.9	88.8	37	*M. avium *
38	9.3	9.3	8.4	9.3	9.3	9.9	7.4	8.4	8.0	9.3	9.3	10.8	9.5	9.5	9.1	9.5	8.4	9.3	9.1	9.1	10.6	10.4	8.9	8.2	9.9	11.5	12.6	12.1	9.7	9.7	9.7	9.7	9.7	10.0	9.9	7.4	6.7	—	94.3	93.7	93.5	93.4	92.4	93.9	93.0	93.7	93.0	89.0	38	*M. malmoense *
39	7.4	7.4	7.0	7.2	7.0	7.2	6.1	5.9	6.3	7.2	7.2	8.4	7.4	7.4	10.2	7.8	7.6	8.0	7.8	7.8	9.7	7.8	7.2	7.4	7.4	10.2	12.3	11.9	8.9	8.9	8.9	8.9	8.9	8.7	8.9	4.5	4.7	5.9	—	94.5	94.1	94.1	93.4	95.1	94.7	94.3	94.3	90.7	39	*M. intracellulare *
40	7.6	7.6	6.7	7.2	7.4	8.4	5.3	6.7	7.6	8.4	8.4	8.4	8.6	8.6	10.1	8.0	7.2	7.6	7.4	7.4	9.7	7.6	7.8	6.7	7.6	9.5	11.5	11.0	8.2	8.2	8.2	8.2	8.2	8.0	8.4	5.5	5.1	6.5	5.7	—	94.3	94.3	94.9	95.1	95.4	93.9	93.4	89.6	40	*M. scrofulaceum *
41	7.8	7.8	7.2	7.4	6.8	8.6	6.5	7.2	7.0	9.5	9.5	8.9	9.1	9.1	11.2	7.8	7.8	8.4	8.2	8.2	10.8	8.2	7.0	7.6	8.7	9.9	11.2	10.8	8.4	8.4	8.4	8.4	8.4	8.7	9.5	6.3	5.7	6.8	6.1	5.9	—	99.6	94.5	96.0	95.6	95.6	93.7	89.6	41	*M. marinum *
42	7.8	7.8	7.2	7.0	6.8	8.2	6.5	7.2	7.0	9.5	9.5	8.9	9.1	9.1	11.0	7.4	7.4	8.4	8.2	8.2	10.6	8.2	6.5	7.4	8.2	9.5	11.2	10.8	8.7	8.7	8.7	8.7	8.7	8.9	9.5	5.9	5.3	7.0	6.1	5.9	0.4	—	94.3	96.0	95.6	95.4	93.9	89.4	42	*M. ulcerans *
43	8.0	8.0	7.6	7.6	7.4	9.1	6.7	7.4	7.2	9.3	9.3	9.5	9.3	9.3	11.0	9.3	8.9	9.3	9.1	9.1	11.2	8.6	8.2	7.4	8.4	11.0	12.6	11.9	8.4	8.4	8.4	8.4	8.4	8.7	10.1	6.1	5.7	8.0	7.0	5.3	5.7	5.9	—	94.3	95.3	95.1	93.4	89.0	43	*M. gordonae *
44	7.0	7.0	6.5	6.7	6.5	7.4	6.3	7.0	6.3	8.6	8.6	8.6	8.9	8.9	11.7	8.0	7.6	8.4	8.2	8.2	9.9	7.8	6.5	7.0	8.0	8.4	10.6	10.1	7.6	7.6	7.6	7.6	7.6	7.8	8.6	5.1	4.7	6.3	5.1	5.1	4.1	4.1	5.9	—	95.6	95.4	94.5	90.1	44	*M. szulgai *
45	7.0	7.0	6.5	6.5	7.0	7.2	5.9	6.7	6.7	9.1	9.1	8.0	8.6	8.6	10.6	6.7	7.6	8.0	7.8	7.8	9.3	7.4	6.3	6.1	7.0	8.9	11.0	10.6	7.0	7.0	7.0	7.0	7.0	7.2	7.4	4.3	4.1	7.4	5.5	4.7	4.5	4.5	4.9	4.5	—	96.4	94.7	88.8	45	*M. asiaticum *
46	8.0	8.0	7.4	7.6	7.4	8.6	7.8	8.0	7.6	10.4	10.4	9.7	9.3	9.3	11.2	9.1	9.1	9.3	9.1	9.1	9.7	8.9	8.0	7.2	8.2	9.9	12.8	12.3	8.2	8.2	8.2	8.2	8.2	8.5	9.1	5.5	4.9	6.5	5.9	6.3	4.5	4.7	5.1	4.7	3.7	—	95.8	88.8	46	*M. kansasii *
47	7.6	7.6	7.6	7.4	8.4	9.1	7.0	8.2	7.6	8.9	8.9	10.8	10.1	10.1	11.9	9.7	9.5	8.4	8.2	8.2	10.8	9.7	8.9	8.0	9.1	10.4	14.2	13.7	8.7	8.7	8.7	8.7	8.7	8.9	9.7	6.5	6.3	7.4	5.9	7.0	6.6	6.3	7.0	5.7	5.5	4.3	—	87.9	47	*M. gastri *
48	11.7	11.7	11.5	11.7	11.2	13.0	10.4	9.7	11.7	11.9	11.9	12.8	11.0	11.0	13.5	12.6	10.6	11.7	11.9	11.9	11.2	11.7	11.9	11.7	12.8	14.2	14.2	13.5	13.0	13.0	13.0	13.0	13.0	13.0	13.5	12.4	12.1	11.9	9.9	11.2	11.2	11.5	11.9	10.6	12.1	12.1	13.2	—	48	*Rhodococcus equi *

	1	2	3	4	5	6	7	8	9	10	11	12	13	14	15	16	17	18	19	20	21	22	23	24	25	26	27	28	29	30	31	32	33	34	35	36	37	38	39	40	41	42	43	44	45	46	47	48		

**Table 3:** Comparison of mycobacteria identified by 16S rDNA/ITS/hsp65/rpoB gene sequencing and by rpsA sequencing among the 188 clinical isolates^a^.

Primary identification^b^ (*n*)	Primary identification^b^ (*n*)			
	First choice		Second choice	
	Identification (*n*)	%match	Identification (*n*)	%match
*M. intracellulare* (75)	*M. intracellulare* (4)	**100%**	*M. avium* (4)	95.6%
	*M. intracellulare* (1)	**99.8%**	*M. avium* (1)	**95.3%**
	*M. intracellulare* (53)	**98.9%**	*M. avium* (53)	**95.1%**
	*M. intracellulare* (10)	**98.7%**	*M. avium* (10)	**94.9%**
	*M. intracellulare* (1)	**98.5%**	*M. avium* (1)	**95.3%**
	*M. intracellulare* (3)	**98.1%**	*M. avium* (3)	**94.7%**
	*M. intracellulare* (1)	**97.5%**	*M. avium* (1)	**93.2%**
	*M. intracellulare* (2)	**97.1%**	*M. avium* (2)	**96.1%**

*M. abscessus* (39)	*M. abscessus* (9)	**100%**	*M. abscessus *subspecies* massiliense* (9)	**98.7%**
	*M. abscessus* (10)	**99.8%**	*M. abscessus *subspecies* massiliense* (10)	**98.5%**
	*M. abscessus *subspecies * massiliense* (20)	**99.4%**	*M. abscessus* (20)	**99.1%**

*M. kansasii* (23)	*M. kansasii* (22)	**100%**	*M. asiaticum* (22)	96.3%
	*M. szulgai* (1)	**96.6%**	*M. marinum/M. ulcerans* (1)	**96.0%**

*M. avium* (10)	*M. avium *subspecies* paratuberculosis* (5)	**99.6%**	*M. avium* (5)	**99.4%**
	*M. avium *subspecies* paratuberculosis* (4)	**99.8%**	*M. avium* (4)	**99.2%**
	*M. avium *subspecies* paratuberculosis* (1)	**97.5%**	*M. avium* (1)	**97.0%**

*M. fortuitum *complex (17)	*M. fortuitum* (2)	**100%**	*M. senegalense/M. thermoresistibile* (2)	98.1%
	*M. fortuitum* (11)	**99.8%**	*M. porcinum* (11)	**98.5%**
	*M. fortuitum* (1)	**99.6%**	*M. porcinum* (1)	**98.3%**
	*M. fortuitum/M. porcinum* (1)	**98.9%**	*M. senegalense/M. thermoresistibile* (1)	**98.3%**
	*M. thermoresistibile/M. senegalense* (2)	**99.6%**	*M. fortuitum* (2)	**98.1%**

*M. gordonae* (7)	*M. gordonae* (1)	**99.2%**	*M. asiaticum* (1)	**95.5%**
	*M. gordonae* (2)	**98.5%**	*M. asiaticum* (2)	**96.3%**
	*M. gordonae* (3)	**95.9%**	*M. asiaticum* (3)	**95.5%**
	*M. szulgai* (1)	**95.9%**	*M. gordonae* (1)	**94.7%**

*M. tuberculosis *complex (16)	*M. tuberculosis* (2)	**100%**	*M. canetti* (2)	**99.8%**
	*M. tuberculosis* (12)	**99.8%**	*M. canetti* (12)	**99.6%**
	*M. tuberculosis* (2)	**99.6%**	*M. canetti* (2)	**99.4%**

*M. neoaurum* (1)	*M. neoaurum* (1)	**98.7%**	*M. smegmatis* (1)	**95.4%**

^a^Numbers in parentheses (*n*) represent the numbers of isolates identified as a particular species.

^b^Identification based on sequencing of at least two of the following: 16S rDNA, 16-23S rRNA gene internal transcribed spacer (ITS), and rpoB and hsp65 genes.
